# Post-Mortem Appearances of a Case in Which Tracheotomy Had Been Performed Three Years Previously

**Published:** 1856-09

**Authors:** Henry Smith


*Post-Mortem Appearances of a Case in which Tracheotomy had been performed three years previously.* By Henry Smith, Esq.

In the Medical Times and Gazette for July 9, 1853, will be found the particulars of a case in which the operation of tracheotomy was performed for urgent dyspnœa, the result of acute inflammation of the larynx. The patient, who was at that time a fine hearty young man, aged 28, recovered from the effects of the operation, but he was not able to dispense with the tube. This circumstance was, of course, a source of considerable anxiety to himself and his friends, although he was comparatively comfortable, and was enabled to attend to his duties; nevertheless, when he made any unusual exertion, or was exposed to cold, he suffered from difficulty of breathing and irritation; he was, however, enabled to pass away many hours together with the tube corked up, and this circumstance induced me to think that it might be dispensed with.

With a view to obtain sanction for withdrawing the tube, the majority of the most eminent Physicians and Surgeons in London were consulted at various times, and the greater number considered that the patient might be able to breathe through the natural aperture. One distinguished Surgeon, however, with whom I had a consultation on more than one occasion strongly urged, both upon the patient and myself, the impropriety of closing the artificial opening, and this advice, coupled with the nervous timidity of the patient, prevailed; the tube was always retained.

I had lost sight of this patient, professionally speaking, for nearly three years after the operation, when, on the morning of June 30, I was hastily summoned to see him with Dr. Tunaley, who had been called to him a few hours previously, in consequence of an attack of dyspnœa with which he had been somewhat suddenly seized, and which was dependant, not upon any mechanical obstruction to the tube, but upon a general congestion of the lungs, at first unattended with any secretion, but in a short time accompanied with a profuse discharge from the air passages. Both Dr. Tunaley and Dr. Watson, who had seen him some few days before this attack, had diagnosed a diseased condition of the lung on the left side; this was now well marked. The symptoms under which the patient now labored were most severe—terrible attacks of asphyxia succeeded one another, and he died from exhaustion in forty-eight hours.

In conjunction with Dr. Tunaley I examined the body the next day, and the following were the post-mortem appearances presented:—

The epiglottis was free from disease, but erect, and could with difficulty be made to close over the larynx. The entrance into this portion of the tube was so contracted that a small crow quill would hardly pass it, and on laying it open, great thickening in the submucous texture of the whole of the interior of the larynx was observed, but there was no disease of the mucous membrane, which was at this point smooth and shining, and presenting no cicatrix of previous ulceration.

The artificial opening was just below the cricoid cartilage, and beyond this the whole of the inner surface of the trachea was not only most intensely inflamed, but at several points it was deeply ulcerated; the inflammation extended to below the bifurcation, and the bronchial tubes were much dilated.

Both lungs were greatly congested, but in addition, the upper and back of the left was rendered almost solid, a great number of tubercles being scattered here and there. On the right side there was less disease, but in the middle lobe a large mass of solid tubercular tissue was observed.

Independent of its intrinsic features of interest, this case is worthy of record, for it was well known to the majority of the more eminent Physicians and Surgeons in London, who were consulted by the patient on various occasions, and some of whom will doubtless recognize it, and will be interested to know the sequel, and the pathological condition of the parts, respecting which there was much discussion during life.

The examination fully reveals the fact, that had this patient survived, the tube must have been worn for life, and yet nearly every one consulted on this point, either recommended that the artificial opening should be closed, or held out strong hopes to the patient that he would some time or another get rid of the tube, and he was only prevented from making the attempt by the opinion of one distinguished Surgeon who strongly insisted upon the danger of such a proceeding. That this advice was correct, the condition of the entrance to the larynx fully demonstrated, still those able men who gave a contrary opinion had good grounds for doing so, for the patient could remain many hours when tranquil, breathing with the tube stopped up. And as during the last few months of the patient's life it was evident that mischief was being set up in the lungs, it was thought that the continued presence of the tube might have caused this. I must confess, that after having had opportunities of seeing the state of the patient under various conditions, and when he was breathing both with and without the use of the artificial opening, I was strongly inclined to agree with the majority of those who urged the patient to remove the tube. Such a case as this will probably not often be met with; under similar circumstances, however, there would always be a difficulty to decide whether the patient might or might not safely dispense with the tube, and it appears to me that the particulars of the case may serve to throw some light upon this point.

There is one circumstance connected with the case which must not be omitted. In the Spring of 1855, Dr. Theophilus Thompson saw this patient, and after making a most thorough examination, diagnosed commencing tubercular deposition in the left lung; moreover, he made the following note with reference to the condition of the windpipe, and with which he was kind enough to furnish me:—

"Very little air seems to pass the glottis; there is a granular state of the fauces; a similar condition in the larynx might explain the reduced passage of air, *except that the sound conveys the idea of simple narrowing, rather than unevenness.*"

The post-mortem appearances show how correct was Dr. Thompson's view.—*Medical Times and Gazette.*

